# Morbidity and Mortality Associated With Pediatric Critical Mediastinal Mass Syndrome

**DOI:** 10.7759/cureus.8838

**Published:** 2020-06-26

**Authors:** Saad Nasir, Rafia Jabbar, Faiza Rehman, Muhammad Khalid, Muhammad Rahil Khan, Anwar Haque

**Affiliations:** 1 Internal Medicine, United Medical and Dental College, Creek General Hospital, Karachi, PAK; 2 Pediatrics, The Indus Hospital, Karachi, PAK; 3 Pediatrics, The Children's Hospital & The Institute of Child Health, Multan, PAK

**Keywords:** pediatric cancers, anterior mediastinal mass, critical, acute lymphoblastic leukemia

## Abstract

Objective

The critical mediastinal mass syndrome (CMMS) is a life-threatening condition and is challenging for physicians. We analyse the clinicopathological profile and outcome of CMMS from a large tertiary-care pediatric oncology center in Pakistan.

Methods

We retrospectively reviewed the medical record of a tertiary-care hospital in Pakistan from April 2017 to September 2019 for all children (1 month-16 years) who presented with an anterior mediastinal mass (AMM). A CMMS case is defined as a child with an AMM presenting with cardiorespiratory compromise and needing intensive care support. Demographic data, clinical profile, pathological diagnosis, and outcome of all such children were recorded. Descriptive statistics were applied using the Statistical Package for the Social Sciences (SPSS), version 22 (IBM Corp., Armonk, NY).

Results

Of the total 221 mediastinal masses, 61 children were diagnosed as CMMS and enrolled in the study. The mean age was 9 ± 3.3 years, and 68.9%% were male; 65.6% of patients had a weight for age less than the fifth percentile. A total of 49.2% of patients had a duration of illness of more than one month before diagnosis. Fever (97.6%) and lymphadenopathy (82%) were the most common findings, along with respiratory and cardiovascular signs and symptoms; 9.8% had superior vena cava syndrome. The pericardial effusion was present in 54.6% and 27.9% had pleural effusion. Peripheral blood flow cytometry made the diagnosis in 59%, peripheral lymph node biopsy in 13%, mediastinal core biopsy in 5%, and pleural fluid flow cytometry in one case; 62.3% had a white blood cell count of >100,000/mm^3^. A total of 72.1% (n=44) cases were diagnosed as T-cell acute lymphoblastic leukemia in our cohort. Clinical and laboratory tumor lysis syndrome developed in 10% and 73% of cases, respectively. Mechanical ventilation was required in 9.8% of the cohort. Mortality was reported in 10 (16.4%) patients.

Conclusion

We found that the 100% fatality rate with controlled positive pressure ventilation and spontaneous breathing is ideal. Tumour lysis syndrome was the most common morbidity in our cohort.

## Introduction

Anterior mediastinal mass (AMM) is common in pediatric age, but the incidence is unknown [1]. AMM in children has varied presentations from asymptomatic to life-threatening events like respiratory arrest or cardiovascular collapse and even death. The most common presentations of childhood AMM are similar to symptoms and signs seen in common respiratory illnesses [2]. Several clinical reports described life-threatening events in AMM in children during treatment, especially related to procedures or postural changes either as acute respiratory or cardiovascular compromise [3,4]. Children are more vulnerable due to a small thoracic cavity size, more compressible cartilaginous structure of the airway, and higher oxygen consumption rate with little reserve [5]. AMM in children is challenging, and sometimes a nightmare situation for pediatric oncologists and pediatric intensivists. A study described one spectrum of childhood AMM presenting with respiratory symptoms and signs or symptoms and signs of superior vena cava syndrome, presence of pleural effusion and/or pericardial effusion or radiological evidences of compression/deviation of the airway or great vessel as critical mediastinal mass syndrome (CMMS) [6]. These cases need a multidisciplinary team approach for diagnostic, therapeutic, and supportive care to improve the outcome and avoid preventable complications to decrease morbidity and mortality [7,8].

Published research on CMMS is limited; hence, we investigated these cases in the present study to determine the mortality and morbidity in CMMS. Our secondary aim was to determine the clinicopathological profile of these patients.

## Materials and methods

A retrospective cohort study was done from April 2017 to September 2019 at the Pediatric Intensive Care Unit (PICU) of a tertiary-care center with a very high burden of pediatric oncology patients, registering more than 800 new cancers in children annually. The study participants included all children (age range: 1 month-16 years) with anterior mediastinal mass presenting acutely with cardiorespiratory symptoms being admitted in PICU. Age, gender, ethnicity, weight, nutritional status, duration of illness along with clinical variables and outcome variables (morbidity [defined complications occurred in PICU], and mortality as death in the same admission) were collected on a structured data collection sheet. Patients confidentiality was maintained. This study aimed to determine the clinicopathological profile and outcomes of CMMS. Ethical approval to conduct the study was obtained from the Institutional Review Board of the Indus Hospital (IRD_IRB_2019_09_012).

CMMS was defined as a mass in the anterior mediastinum that causes respiratory signs and symptoms, signs of superior vena cava syndrome, radiological evidence of compression, deviation of the airway, or great vessel or presence of pleural/pericardial effusion [6]. Hyperleukocytosis was defined as a peripheral white blood cell count greater than 100,000/mL. Morbidity was defined as a respiratory failure (use of mechanical ventilation), sepsis (empirical use of broad-spectrum antibiotics [vancomycin, meropenem, and colistin] with clinical suspicion of sepsis), shock (use of any inotrope or vasopressor), acute kidney injury (rise in serum creatinine based on Kidney Disease: Improving Global Outcomes [KDIGO] criteria) and tumor lysis syndrome (laboratory or clinical based on Bishop’s criteria) [9,10].

Data were entered into and analysed using the Statistical Package for the Social Sciences (SPSS), version 22 (IBM Corp., Armonk, NY). Means and standard deviations were calculated for continuous variables such as age, laboratory characteristics, and length of hospital stay. Frequency and proportions were used for categorical variables such as gender, and clinical and laboratory characteristics. Simple descriptive statistics were applied. Univariate logistic regression analysis was performed to assess the ability of variables such as age, gender, nutritional status, duration of symptoms, diagnosis, and laboratory findings including, total leukocyte count and platelet count, to predict the mortality outcome.

## Results

Of the total 221 mediastinal mass cases, 61 (27.6%) children were enrolled during the study period. The mean age was 9 ± 3.34 years, and there was a male predominance (68.9%); 65.6% (n=40) of patients had a weight for age less than the fifth percentile, while the mean weight was 23.2 ± 10 kg. Approximately half of the patients had the illness for one month before getting diagnosed with this condition. Other demographic features are mentioned in Table 1.

**Table 1:** Demographic characteristics of children presenting with critical mediastinal mass (n=61) n, sample size.

Variable	n (%)
Age groups	
2-5 years	11 (18.0)
6-10 years	33 (54.1)
>10 years	17 (27.9)
Gender	
Male	42 (68.9)
Female	19 (31.1)
Ethnicity	
Sindhi	31 (50.8)
Punjabi	01 (1.6)
Balochi	18 (29.5)
Not known	11 (18.0)
Weight (kg) mean ± SD	23.2 ± 10
Malnourished	
Yes	40 (65.6)
No	21 (34.4)
Duration of illness	
<1 week	08 (13.1)
1-2 weeks	09 (14.8)
>2-3 weeks	03 (4.9)
>3-4 weeks	11 (18.0)
>4 weeks	30 (49.2)
Length of stay in days (mean ± SD)	6 ± 5.23 (range 1-32)

Most of these children presented with nonspecific symptoms such as fatigue (63.9%), followed by weight loss, respiratory distress, cough, while fever (96.7%) and lymphadenopathy (82%) were the most frequent clinical signs present (Table 2). Six (9.8%) patients had superior vena cava syndrome.

**Table 2:** Clinical characteristics of children presenting with critical mediastinal mass (n=61) ^a^Other symptoms include chest pain, dysphagia, night sweats, and wheeze. ^b^Other clinical signs include facial swelling, upper body edema, stridor, ronchi, and decreased level of consciousness. n, sample size; SVCS, superior vena cava syndrome.

Clinical characteristic	n (%)
Presenting symptoms	
Cough	16 (26.2)
Respiratory distress	18 (29.5)
Weight loss	23 (37.7)
Fatigue	39 (63.9)
Headache	13 (21.3)
Others^a^	07 (11.5)
Clinical signs	
Fever	59 (96.7)
Tachypnea	44 (72.1)
Decrease air entry	20 (32.8)
Crepitations in lungs	15 (24.6)
Desaturation	24 (39.3)
Lymphadenopathy	50 (82.0)
Hepatosplenomegaly	46 (75.4)
SVCS	06 (9.8)
Other clinical signs^b^	19 (31.2)

Our data showed the presence of hyperleukocytosis in 38 (62.3%) patients, while more than half of the children had positive findings on chest X-ray, including pleural effusion in 17 (27.9%) patients. Echocardiography done in 55 patients showed pericardial effusion in 54.6% of patients, while three cases had partial great vessel obstruction (Table 3).

**Table 3:** Laboratory characteristics of children presenting with critical mediastinal mass (n=61) n, sample size; LDH, lactate dehydrogenase.

Laboratory characteristics	n (%)
Hematology profile	
Hemoglobin (g/dL), (mean ± SD)	7.38 ± 2.70
Platelet counts	
<50,000 (per mm^3^)	41 (67.2)
50,000-100,000 (per mm^3^)	06 (9.8)
>100,000 (per mm^3^)	14 (23.0)
Leucocyte counts	
<10,000/mL	05 (8.2)
10,000-50,000/mL	16 (26.2)
51,000-100,000/mL	02 (3.3)
>100,000/mL	38 (62.3)
Deranged coagulation profile	10 (16.4)
Positive smear for blast cells	49 (80.3)
Serum potassium (mmol/L) (mean ± SD)	3.97 ± 1.03
Serum uric acid (mg/dL) (mean ± SD)	7.90 ± 4.10
Serum LDH (mini-max)	361-5646
Serum creatinine (mg/dL) (mean ± SD)	0.5 ± 0.24
Serum phosphate (mg/dL) (mean ± SD)	3.75 ± 0.91
Alanine aminotransferase (mean ± SD)	42.85 ± 54.39
Positive blood culture	07 (11.5)
Radiological profile	
Chest X-ray performed	61 (100)
Effusion on chest X-ray	17 (27.9)
Lung abnormalities	19 (31.1)
CT-scan chest performed	08 (13.1)
Effusion on CT chest	03 (37.5)
Tracheal compression	01 (1.6)
Vessel compression	02 (3.2)
Echocardiography done	55 (90.2
Pericardial effusion	30 (54.6)
Vessel compression	03 (5.5)
Ejection fraction (min-max)	50-87

**Figure 1:**
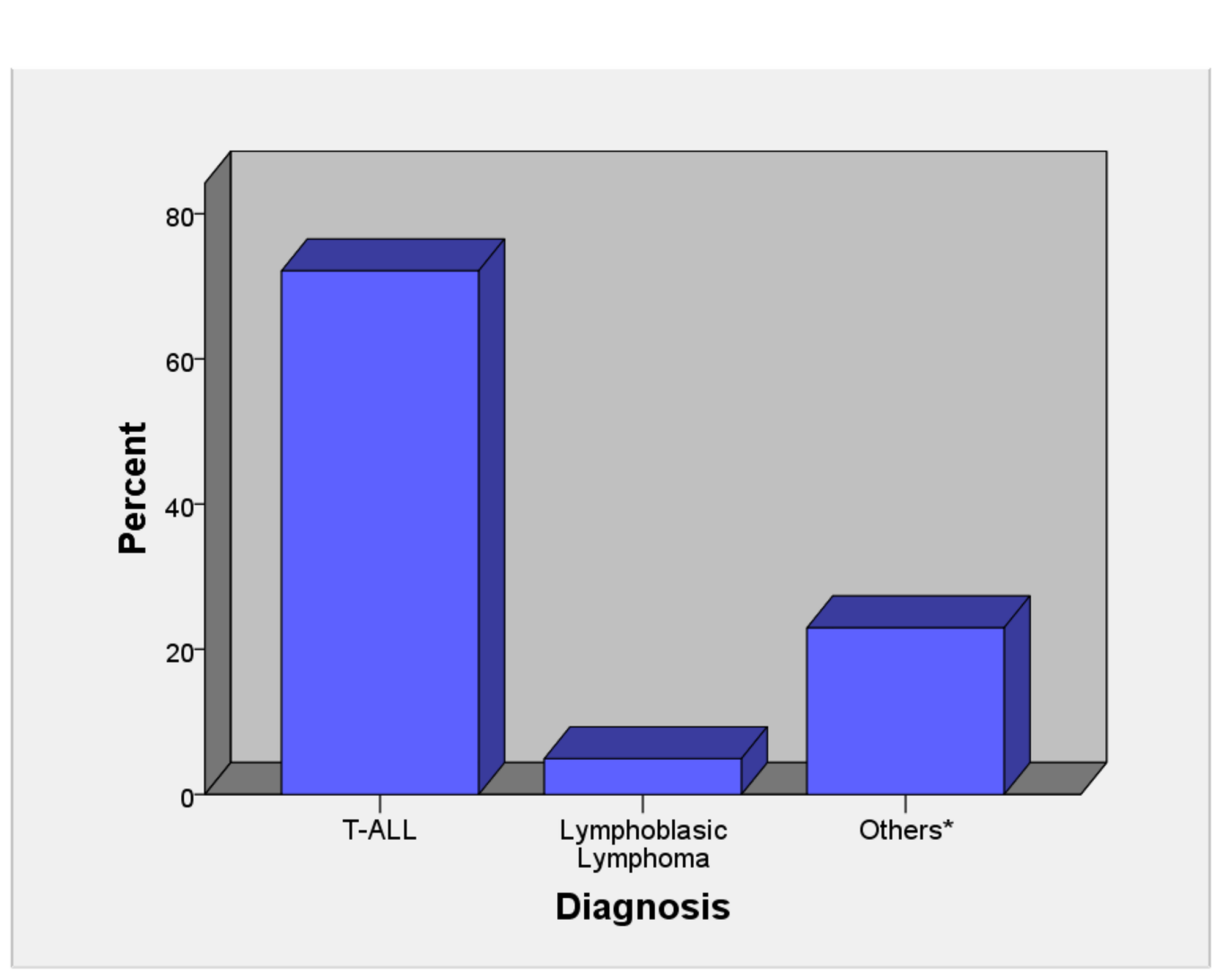
Diagnosis in patients with anterior mediastinal masses (n=61) *Neuroblastoma, Ewing sarcoma, B-cell non-Hodgkin’s lymphoma, suspected leukemia died before diagnosis, and suspected Hodgkin’s lymphoma. T-ALL, T-cell acute lymphoblastic leukemia.

Figure 1 shows that the most common diagnosis was T-cell acute lymphoblastic leukemia (72.1%), while 4.4% had lymphoblastic lymphoma. Peripheral blood flow cytometry was used to make the diagnosis in most (59%) of these children, followed by peripheral lymph node biopsy (13.1%). A total of 22 (32.6%) patients developed tumor lysis syndrome as a complication. The mean length of hospital stay was 6 ± 5.2 days. Analysis of the outcomes revealed that mortality was observed in 10 (16.4%) patients, while all six patients in our cohort, who were put on mechanical ventilation, did not survive (Table 4).

**Table 4:** Mode of diagnosis, intervention, and outcome of children presenting with critical mediastinal mass (n=61) n, sample size; TLS, tumor lysis syndrome; LP, lumbar puncture.

Characteristic	n (%)
Mode of diagnosis	
On peripheral blood flow cytometry	36 (59)
On pleural fluid flow cytometry	01 (1.6)
On mediastinal mass core biopsy	03 (4.9)
On peripheral lymph node biopsy	08 (13.1)
Complications	
Septic shock	13 (21.3)
Acute kidney injury on admission	05 (8.2)
Acute kidney injury during treatment	07 (11.5)
TLS	22 (36.1)
Clinical TLS	02 (9.1)
Laboratory TLS	16 (72.7)
Both clinical and laboratory TLS	05 (22.7)
Intervention	
Cytoreduction	60 (98.4)
Chemotherapy	35 (58.3)
Both chemotherapy and LP	25 (41.7)
Pre-treatment steroids	04 (6.6)
Inotropic support	08 (13.1)
Mechanical ventilation	06 (9.8)
Dialysis	02 (3.3)
Cardiopulmonary resuscitation	04 (6.6)
Outcome	
Mortality	10 (16.4)

A univariate logistic regression analysis was calculated to predict participants' outcomes based upon other variables such as age, gender, nutritional status, duration of symptom, diagnosis, total leukocyte count, platelet count, and mechanical ventilation. We found a non-significant relationship between all the other predictor values (p-value >0.05), except mechanical ventilation and the mortality outcome (p-value <0.001) (Table 5).

**Table 5:** Univariate logistic regression analysis for determinants of mortality in children presenting with critical mediastinal mass (n=61) n, sample size; T-ALL, T-cell acute lymphoblastic leukemia.

Determinant	Odds ratio	95% CI	p-value
Age (yrs)	0.81	0.65-1.00	0.05
Gender			
Male	1	-	-
Female	1.07	0.24-4.67	0.93
Malnourished			
No	1	-	-
Yes	0.79	0.18-3.41	0.75
Duration of symptoms			
Up to 2 weeks	1	-	-
>2-4 weeks	0.54	0.09-2.94	0.47
>4 weeks	1.93	0.34-10.83	0.46
Diagnosis			
Others	1	-	-
T-ALL	3.25	0.80-13.16	0.09
Total leucocyte count			
<100,000/mL	1	-	-
≥100,000/mL	0.79	0.18-3.42	0.75
Platelet count			
>100,000 (per mm^3^)	1	-	-
<100,000 (per mm^3^)	0.81	0.15-4.36	0.81
Mechanical ventilation			
Yes	2.5	1.17-5.34	<0.001
No	1	-	-

## Discussion

This is the first comprehensive report on children with CMMS from a PICU of a lower middle-income country. We found that the mortality rate was 16.4% (n=10) in children with CMMS. The compressive effects of these masses on the airways or the great vessels owing to small intra-thoracic volume in young children can lead to cardiorespiratory compromise, resulting in death. Various studies report 25%-45% mortality in these patients [11,12].

T-cell acute lymphoblastic leukemia was the most common malignancy (72%, n=44) in our cohort of CMMS. Kashif et al. found T-cell acute lymphoblastic leukemia in 35% of children with a malignant mediastinal mass and found it to be the strongest risk factor for mortality (p <0.001) [11]. The reported mortality was 45% in their report. In another report from India, Mathan and Ananthamurthy described 53.3% of T-cell acute lymphoblastic leukemia in their children with mediastinal mass [13]. Similarly, Ravindranath et al. observed very high mortality in acute lymphoblastic leukemia with a mediastinal mass [14]. However, the prognostic significance of mediastinal mass in T-cell acute lymphoblastic leukemia is controversial. We did not find any association of mortality with primary diagnosis in any study.

The most striking clinical observation of this report is that the controlled positive pressure ventilation with muscle paralysis in these patients was associated with 100% mortality (p <0.001). The positive pressure ventilation with neuromuscular blockade is detrimental, further aggravating airway obstruction and leading to cardiovascular collapse from fatal airway obstruction, which is not amenable to routine cardiopulmonary resuscitation in the absence of rigid bronchoscopy and extracorporeal life-support system. There are multiples reasons for fatal airway obstruction in children with CMMS, including small lung volume, loss of spontaneous diaphragmatic activity, decrease in chest wall muscle tone, the position of the patient, and compression effect of the tumor on airway distal to the endotracheal tube. Previously published reports demonstrated that spontaneous ventilation is ideal in such patients [15-17].

The most important dilemma of determining the primary diagnosis in such life-threatening cases is challenging and needs a multidisciplinary team approach like anesthesia, interventional radiologist, and pediatric thoracic surgeon. In most cases, the alternative tissue was used for primary diagnosis and rarely needed mediastinal tissue biopsy as in many clinical reports on symptomatic mediastinal mass in children. In our study, we established the primary diagnosis from alternative tissue: 59% from peripheral blood flow cytometry and 13% from cervical lymph node biopsy. Hence, mediastinal tissue biopsy was needed in only 5% of cases. Garey et al. in their study diagnosed all patients with T-cell acute lymphoblastic leukemia on either peripheral blood smear (50%) or bone marrow aspiration (50%) [7]. Acker et al. included 69 children with an anterior mediastinal mass and most cases (81%) needed a biopsy tissue from outside the mediastinum with minimal sedation and spontaneous breathing [18].

Clinically, children with mediastinal masses are often symptomatic of both respiratory and constitutional symptoms because of malignancy. They are treated initially for common respiratory illnesses like pneumonia and asthma [12]. Lam et al. analysed clinical records of 29 children with mediastinal mass, and an anteriorly located mass was a significant factor to develop respiratory compromise (p=0.019) [6]. A total of 24.1% of children were asymptomatic at the time of diagnosis. Symptomatic children presented with cough (33.3%), dyspnea (23.8%), and chest pain (19%), while one patient developed wheeze [6]. Our results show that most children had non-specific symptoms such as fatigue, weight loss, and cough while fever and tachypnea were the most common clinical manifestations. These results are comparable to other studies [6,12,19,20]. The demographic analysis showed the mean age at diagnosis to be 9 years, higher than that seen in the existing literature [11,21]. Among the patients in this study, tumor lysis syndrome was the most common (36.1%) followed by septic shock (21%) and acute kidney injury (11%).

This study comprehensibly reviews the PICU course of CMMS in children and further strengthens the existing literature. Study limitations included retrospective data collection from a single institution and a small sample size.

## Conclusions

We found that the 100% fatality rate with controlled positive pressure ventilation and spontaneous breathing is ideal. Tumor lysis syndrome was the most common morbidity in our cohort. The rapid use of alternative tissue for primary diagnosis in most cases and the early initiation of intensive chemotherapy may improve the outcome in these children.

## References

[1] Pearson JK, Tan GM (2015). Pediatric anterior mediastinal mass: a review article. Semin Cardiothorac Vasc Anesth.

[2] Saraswatula A, McShane D, Tideswell D, Burke GA, Williams DM, Nicholson JC, Murray MJ (2009). Mediastinal masses masquerading as common respiratory conditions of childhood: a case series. Eur J Pediatr.

[3] Piastra M, Ruggiero A, Caresta E, Chiaretti A, Pulitano S, Polidori G, Riccardi R (2005). Life-threatening presentation of mediastinal neoplasms: report on 7 consecutive pediatric patients. Am J Emerg Med.

[4] Perger L, Lee EY, Shamberger RC (2008). Management of children and adolescents with a critical airway due to compression by an anterior mediastinal mass. J Pediatr Surg.

[5] Kar SK, Ganguly T, Dasgupta CS, Goswami A (2014). Cardiovascular and airway considerations in mediastinal mass during thoracic surgery. J Clin Exp Cardiolog.

[6] Lam JC, Chui CH, Jacobsen AS, Tan AM, Joseph VT (2004). When is a mediastinal mass critical in a child? An analysis of 29 patients. Pediatr Surg Int.

[7] Garey CL, Laituri CA, Valusek PA, St Peter SD, Snyder CL (2011). Management of anterior mediastinal masses in children. Eur J Pediatr Surg.

[8] Malik R, Mullassery D, Kleine-Brueggeney M, Atra A, Gour A, Sunderland R, Okoye B (2019). Anterior mediastinal masses - a multidisciplinary pathway for safe diagnostic procedures. J Pediatr Surg.

[9] Fujii T, Uchino S, Takinami M, Bellomo R (2014). Validation of the kidney disease improving global outcomes criteria for AKI and comparison of three criteria in hospitalized patients. Clin J Am Soc Nephrol.

[10] Cairo MS, Bishop M (2004). Tumour lysis syndrome: new therapeutic strategies and classification. Br J Haematol.

[11] Kashif RU, Faizan M, Anwar S (2019). Pediatric malignant mediastinal masses. J Coll Physicians Surg Pak.

[12] Lerman J (2007). Anterior mediastinal masses in children. Semin Anesth Perioperat Med Pain.

[13] Mathan L, Ananthamurthy A (2018). Clinicopathological attributes of T-lymphoblastic lymphoma seen in a tertiary care centre. Clin Cancer Investig J.

[14] Ravindranath Y, Kaplan J, Zuelzer WW (1975). Significance of mediastinal mass in acute lymphoblastic leukemia. Pediatrics.

[15] Tütüncü AC, Kendigelen P, Kaya G (2017). Anaesthetic management of a child with a massive mediastinal mass. Turk J Anaesthesiol Reanim.

[16] Williams A, Singh G, George SP (2015). Procedural sedation for a child with a mediastinal mass and superior vena caval syndrome. J Anaesthesiol Clin Pharmacol.

[17] Stricker PA, Gurnaney HG, Litman RS (2010). Anesthetic management of children with an anterior mediastinal mass. J Clin Anesth.

[18] Acker SN, Linton J, Tan GM (2015). A multidisciplinary approach to the management of anterior mediastinal masses in children. J Pediatr Surg.

[19] Chen CH, Wu KH, Chao YH, Weng DF, Chang JS, Lin CH (2019). Clinical manifestation of pediatric mediastinal tumors, a single center experience. Medicine (Baltimore).

[20] Mushtaq N, Alam MM, Aslam S, Fadoo Z, Anwar ul H (2014). Malignant mediastinal mass in children: a single institutional experience from a developing country. J Pak Med Assoc.

[21] Gun F, Erginel B, Unuvar A, Kebudi R, Salman T, Celik A (2012). Mediastinal masses in children: experience with 120 cases. Pediatr Hematol Oncol.

